# The rise of health biotechnology research in Latin America: A scientometric analysis of health biotechnology production and impact in Argentina, Brazil, Chile, Colombia, Cuba and Mexico

**DOI:** 10.1371/journal.pone.0191267

**Published:** 2018-02-07

**Authors:** Dante Israel León-de la O, Halla Thorsteinsdóttir, José Víctor Calderón-Salinas

**Affiliations:** 1 Doctorate Program on Science, Technology and Society, Centro de Investigación y de Estudios Avanzados del Instituto Politécnico Nacional, Mexico City, Mexico; 2 Institute of Health Policy, Management and Evaluation, University of Toronto, Toronto, Ontario, Canada; 3 Small Globe Incorporated, Toronto, Ontario, Canada; 4 Biochemistry Department, Centro de Investigación y de Estudios Avanzados del Instituto Politécnico Nacional, Mexico City, Mexico; Universidade de Brasilia, BRAZIL

## Abstract

This paper analyzes the patterns of health biotechnology publications in six Latin American countries from 2001 to 2015. The countries studied were Argentina, Brazil, Chile, Colombia, Cuba and Mexico. Before our study, there were no data available on HBT development in half of the Latin-American countries we studied, i.e., Argentina, Colombia and Chile. To include these countries in a scientometric analysis of HBT provides fuller coverage of HBT development in Latin America. The scientometric study used the Web of Science database to identify health biotechnology publications. The total amount of health biotechnology production in the world during the period studied was about 400,000 papers. A total of 1.2% of these papers, were authored by the six Latin American countries in this study. The results show a significant growth in health biotechnology publications in Latin America despite some of the countries having social and political instability, fluctuations in their gross domestic expenditure in research and development or a trade embargo that limits opportunities for scientific development. The growth in the field of some of the Latin American countries studied was larger than the growth of most industrialized nations. Still, the visibility of the Latin American research (measured in the number of citations) did not reach the world average, with the exception of Colombia. The main producers of health biotechnology papers in Latin America were universities, except in Cuba were governmental institutions were the most frequent producers. The countries studied were active in international research collaboration with Colombia being the most active (64% of papers co-authored internationally), whereas Brazil was the least active (35% of papers). Still, the domestic collaboration was even more prevalent, with Chile being the most active in such collaboration (85% of papers co-authored domestically) and Argentina the least active (49% of papers). We conclude that the Latin American countries studied are increasing their health biotechnology publishing. This strategy could contribute to the development of innovations that may solve local health problems in the region.

## Introduction

Biotechnology has been lauded to be a panacea that has the potential to contribute to solving problems in diverse fields. In health it has been harnessed to develop health products, such as vaccines to prevent viral diseases, for example, the dengue fever; antibiotics to heal people from bacterial infections, such as tuberculosis; and molecular treatments against diverse types of cancers [1,2]. Biotechnology firms are having impacts on international markets and the sector as a whole generates large incomes to economies of various countries [3–7]. Biotechnology does not only contribute to the economic welfare of high-income countries but is also increasing earnings in emerging economies such as in India and China [4,7–10].

Countries that started to promote biotechnology in the last century have a leading role in the field. The United States (U.S.) is the indisputable global leader in biotechnology. In 2006, the U.S. invested US$ 22 billion exclusively in biotechnology research and development (R&D) and the investment continued to rise and reached US$ 33.9 billion in 2015 [11,12]. In comparison, the second largest investor, Europe as a whole, invested in 2006 US$ 3.6 billion in biotechnology and by 2015 US$ 6.2 billion [11,12]. At the same time, the U.S. Congress was one of the first to introduce biotechnological policies and laws [13]. Other historical examples include the European Union (known as the European Community at the time) that allocated more than US$ 300 million to biotechnology-related programs in the early 1980s [14]. These programs constituted the first step to create the biotechnology industry in Europe. At the time the United Kingdom, Germany and France were the countries with the highest investments in the field outside of North America [15,16]. In 2015, the financing of the global biotechnology industry had reached US$ 180 billion and had almost doubled compared to the 2014 financing of US$ 102.57 billion [17]. This reflects the high expectations and financial attractiveness perceived of biotechnology in the business world and by governments worldwide.

Some countries in Latin America have invested in specific biotechnology fields, such as health, with the objective that the knowledge gained can result in innovations and economic growth [18,19]. For example, the Brazilian government’s investment in biotechnology for the period 2008–2010 was US$1 billion [20]. By 2011 the Brazilian biotechnology companies generated estimated annual gross revenue of over US$1.2 billion [21]. Mexico is another example of a country that has placed economic importance on the biotechnology industry. Its business sector invested in 2011 US$93.9 million in biotechnology R&D [22].

**Fig 1:**
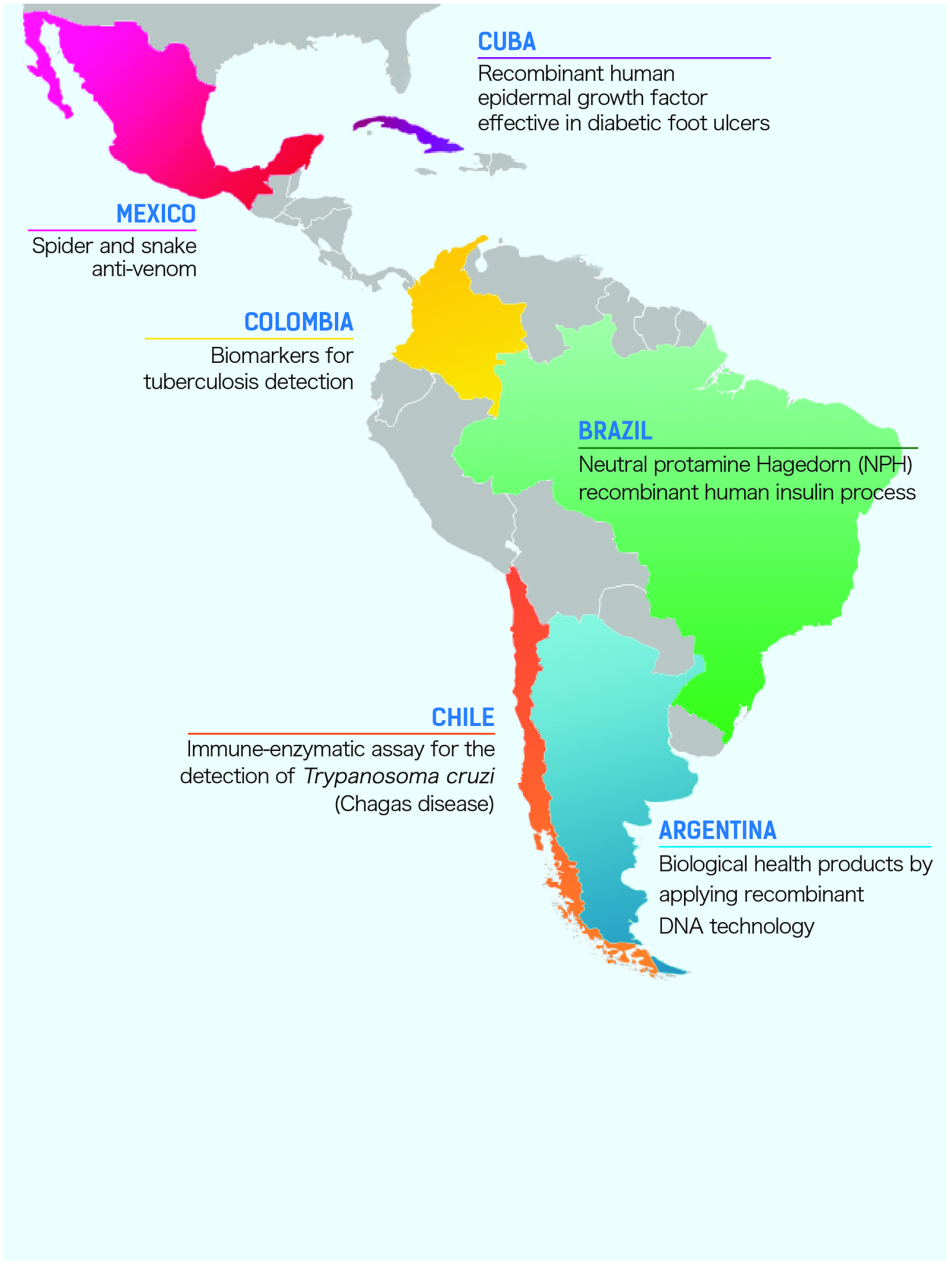
Successful cases of Latin American health biotechnology investment. The expectation of HBT in Latin American countries is not only to generate economic benefits but also to solve local health problems.

Health biotechnology (HBT) is an important biotechnology field on the global market and represented 60% of the biotechnology global market value in 2012 [23]. The expectation of HBT in low-and-middle-income countries (LMICs) is not only to generate economic benefits but also to solve local health problems that are not prioritized by high-income countries [24]. There are several successful cases of Latin American HBT investment (Fig 1). For example, Argentina’s domestic industry has synthesized biological health products by applying recombinant DNA technology. Another example is the neutral protamine Hagedorn (NPH) recombinant human insulin process developed and patented by Brazilian universities and biopharmaceutical firms that can address the type 2 diabetes prevalence in Latin American countries [25,26]; Chile has commercialized an immune-enzymatic assay for the detection of serum antibodies against *Trypanosoma cruzi* (Chagas disease) and other biotechnology clinical assays for tropical diseases [27,28]; Colombia is evaluating a diagnostic assay that uses biomarkers for tuberculosis detection [29]; Cuba has made several innovations in HBT including a new-to-the-world vaccine against type-B bacterial meningitis [30]; the Heberprot-P^®^, a treatment that uses recombinant human epidermal growth factor (EGF) and is effective in several types of diabetic foot ulcers of ischemic and neuropathic type [31]; and Vaxira, an anti-idiotypic cancer vaccine for the treatment of advanced non-small cell lung carcinomas [32]. Lastly, Mexico has developed spider and snake anti-venoms [33]. The antidote may reduce the 32 thousand yearly deaths by attacks from spiders and snakes in Mexico [34,35].

All these innovations have been made possible by the important contribution of scientific knowledge [36]. Nevertheless, there is a lack of information about the levels and main characteristics of the production of scientific knowledge in HBT in Latin America that is necessary to understand the best way to harness the technology for the region. Previous studies have examined HBT development in some of the countries we studied. HBT development in Brazil and Cuba was, for example, studied for the 1991–2002 period. This paper represents an update in data on HBT development in those two countries as well as an opportunity to compare their development with other LA countries where HBT publications have not been studied before. A study of HBT publications would provide a quantifiable approach to study the development of this field and allow comparisons and benchmarking among countries. This information can identify potential strengths and weaknesses in the biotechnology knowledge production that scientific communities and policy makers should pay attention to, and address accordingly, to encourage growth and strengthening of health biotechnology research and industry in Latin America.

To fill this gap we conducted a scientometric analysis of HBT publications in six Latin American countries: Argentina, Brazil, Chile, Colombia, Cuba and Mexico. The questions we are addressing in this paper are as follows:

1. What are the trends in knowledge production in these countries in HBT?
2. Who are the main actors responsible for the scientific-publishing in HBT in the Latin American countries?
3. What are the main research subfields of the focal countries in HBT?
4. What is the emphasis on international and domestic collaboration in the countries under study and what are the main countries the focal nations in this study collaborate with in HBT?

## Methods

The first challenge in this research was to classify HBT. We chose the Biotechnology Statistics Classification used by the Organization for Economic Co-operation and Development (OECD) that is: Health, agro-food, and industry-environmental biotechnology [37]. This OECD classification has attempted to develop categories that are comparable across all countries. We proceeded to delimit what type of biotechnology knowledge would encompass HBT, as well as to identify the initial keywords to conduct the data search. The data search criteria were supported by operationalizing the HBT definition of OECD: “The use of knowledge on cell functions and genetics at the molecular level, including an understanding of deoxyribonucleic acid (DNA), ribonucleic acid (RNA), proteins and enzymes, to develop new therapeutics and diagnostics” [38]. This definition gave us the initial keywords we used to identify new keywords that are related to HBT.

The scientometric analysis was based on data extracted from the Web of Science^®^ (WOS) Core Collection. It has more than 55 million records of peer-reviewed journals and conference proceedings on science in the world [39] and it also has access to Scielo database (Scientific Electronic Library Online). This is a Brazilian database of open access journals in Latin America, Spain, Portugal, the Caribbean and South Africa [40]. Lastly, we used the tools and retrieving algorithms on international citation indices that WOS provides: the citations index; the rank of countries in a field; the science-field classification; the collaboration among countries in a specific field; and the document type classification [41].

We analyzed papers that fit the HBT definition, from the WOS database, with the following inclusion criteria: within the Science Technology domain; published between 2001 and 2015; with at least one author affiliated to an institution from Argentina, Brazil, Chile, Colombia, Cuba or Mexico. We considered articles, abstracts, meeting abstracts and reviews [42,43]. Review articles were included because they represent analytical organization of the HBT knowledge and because WOS at times misclassifies original research articles as review articles and excluding them would contribute to underestimating the HBT knowledge production in the Latin American countries we studied [44–46] Review articles were rare and included about 5% of the articles.

We chose papers using the keywords associated with the HBT definition (Table 1), and selected those that dealt with health research fields compatible with the OECD HBT definition. WOS’s field classification methodology applies the OECD Category scheme. This scheme is supported by the Revised Field of Science and Technology (FOS) Classification from the Frascati Manual 2002 [47], and categorizes its data into six main categories: natural sciences; engineering and technology; medical and health sciences; agricultural sciences; social sciences and humanities [48]. These six categories are then subdivided into approximately 250 subfields [49]

**Table 1:** Keywords used to search WOS for health biotechnology.

	Keywords
A	Antibod*, antigen*, amino acid, antisense,
B	Bio*, Bacter*
C	Cell/, Clon/
D	DNA, Diagnos*
E	ELISA, Enzym*
F	Fung*, Fermen*
G	Gen, Gene, Genome, GMO.
H	Hormo*, Human, Hybrid*
I	Immu*, Interferon
L	Lipid*
M	Molecul*, Metabol*, Mapping, Muta*
O	Organ*, Oligo*
P	PCR, Plasmid$, Phage, Pathway, Primer$, Prote*, pharma*, peptid*, Polymerase.
R	RNA*, Regenera*, Recombi*, Ribonu*
S	Serologic*, Steroid*, Sequenc*
T	Therap*, Tissue$, Transcript*.
V	Viru$, viral$, vaccin*, Vector$
Y	Yeast$

Source: OECD [38] and author´s keywords identifications.

^$^, ^/^ and * are research wildcards used in WOS to find related words

We excluded non-biological health research fields such as biophysics, radiology nuclear medicine, medical imaging, health care sciences services and disease treatment by non-biological methods. Papers that dealt with HBT but focused on social sciences, art humanities, education, bioethics, laws studies and related topics were also excluded. Duplications in each country were identified and deleted using the software QDA miner with Wordstat.

In Table 1, we list the main keywords we used in the WOS searches on the ‘titles’ of the documents. The validation of the database was made by data mining with QDA miner.

## Results and discussion

### Trends in health biotechnology knowledge production in the Latin American countries

#### Levels of publication in health biotechnology

We examined the levels of publication in HBT in the Latin American countries studied. The total amount of HBT publications in the world from 2001–2015 was about 400,000 papers and it represented 1.2% of the world publications in the WOS database at the beginning of 2016. The proportion of HBT papers versus the total papers has decreased by 0.5% during the period of this study. This happened because the rhythm in publications in the other fields was increasing faster than in HBT. This tendency has existed for some years as Thorsteinsdóttir *et al*. (2006) reported a reduction of 0.2% in HBT papers from an earlier period (1991–2002). From these about 400,000 papers in HBT world-wide, 2.6% were authored by the six Latin American countries in this study. This reflects a relatively important participation of the Latin American countries in the field.

**Fig 2:**
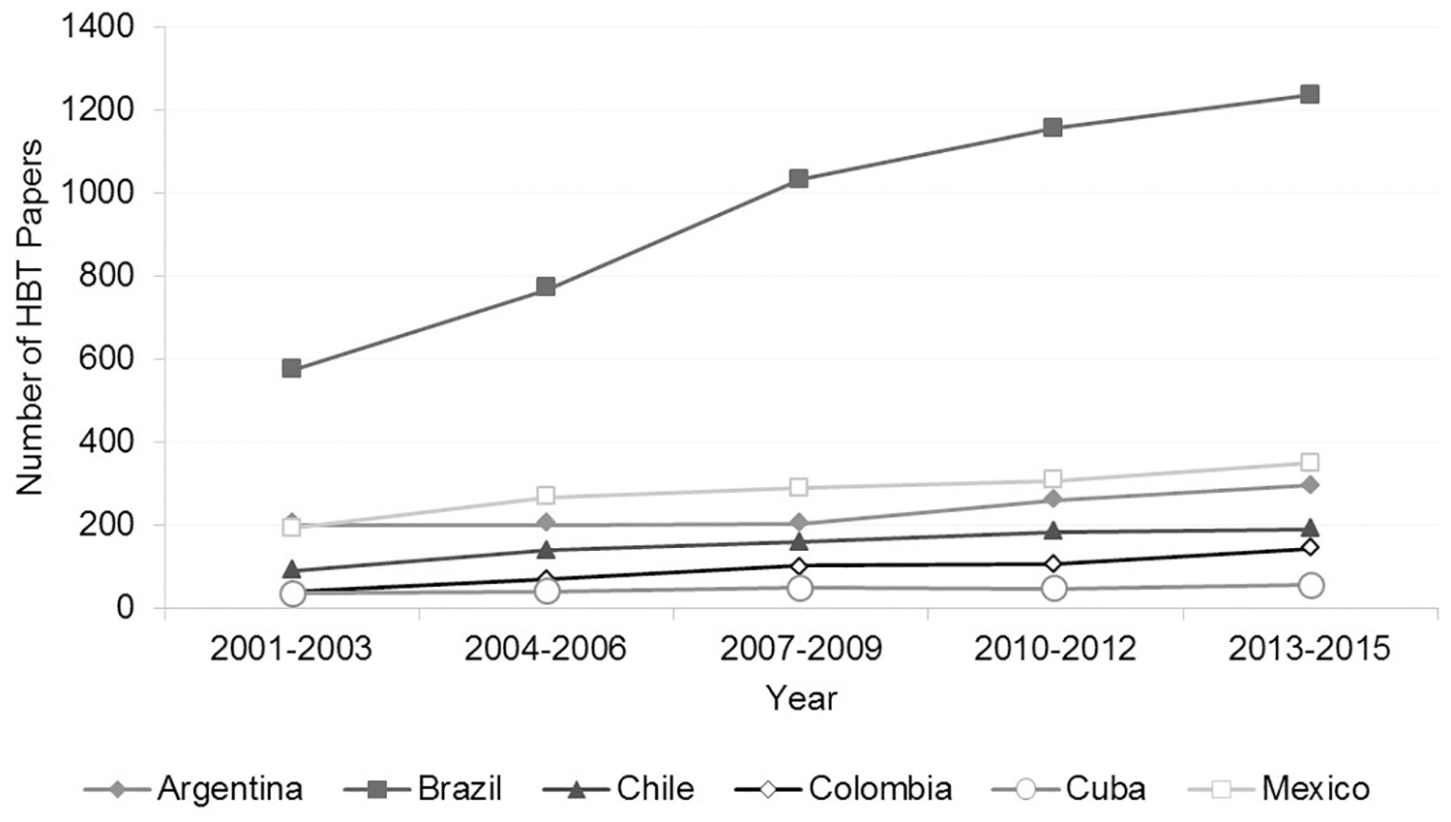
Number of papers in health biotechnology by selected countries, 2001–2015. Source: WOS data [52].

Fig 2 presents the number of papers published by the selected Latin American countries in HBT from 2001–2015. We aggregated the number of papers published for three years to better observe potential trends in knowledge production. To test if the growth in the number of publications of each country was statistically significant, we applied simple linear regression analyses to the data from each country (Fig 2), using the R statistical language [50]. We used the T-Test to check whether the slopes obtained from the equations of the line were significantly different from zero (zero, in this case, means no growth). The significance level was p<0.05 [51]. The statistical analysis shows that the six Latin American countries had a significant growth in HBT papers during the period studied.

The Latin American countries in this study published less than 600 papers in HBT for the first three years period (Fig 2). Most of them published less than 200 papers, with the exception of Brazil, which published 575 papers in HBT. In the early 2000s publishing in HBT seemed to be taking off in three of the Latin American countries: Brazil, Mexico and Chile, whereas Argentina, Colombia and Cuba had almost no change in the levels of their HBT production. Around the mid 2000s, all the countries had increased their publishing in HBT with the exception of Argentina, which has been stuck from 2001–2009 with an average of around 200 papers per three year period. Brazil increased its publications by over one hundred papers in the years 2007–2009 and its rate of publishing has been steadily increasing. At the end of the period of our study, Brazil continued to experience growth in publishing rate, whereas Mexico’s and Colombia’s publications increased less intensively. Argentina’s production started to rise from the 2010–2015 period. Cuba and Chile appear to be in a plateau in terms of HBT publications for the 2007–2015 period.

Brazil has maintained its leadership in publishing in HBT over the other Latin American countries since the beginning of the period studied. Its HBT publications increased 380% by the end of the period. It is well known that funding is necessary for scientific knowledge production [53] as well as the generation of science policy and the promotion of scientific collaboration [54]. Brazil’s relatively high publishing level is partly the result of the government’s early start in promoting biotechnology, in the beginning of the 1980s. For example, the government promoted research in biotechnology by implementing the PRONAB (Programa Nacional de Biotecnologia) in 1982. In the same year Brazil’s government promoted biotechnology research at public universities [55] and in 1985 it started to promote links between public research with the private sector [25]. Another important factor that could explain Brazil’s high production is its relatively high Gross Domestic Expenditure on R&D (GERD) as a percentage of GDP (Gross Domestic Product). The Brazilian government has gradually increased its GERD from 1.00% to 1.2% of its GDP in the years 2000–2012 and has kept it at an average of 1.12% since 2010 to 2013 [56]. Brazil’s GERD was the highest of all the six focal countries.

Mexico ranked second in HBT publishing of the countries examined. However, Mexico’s production did not exceed 400 papers in any of the periods studied, while Brazil published more than 1200 papers at the end of the 2013–2015 period. From 2001 to 2015, Mexico had increased its HBT publications by 233%. In 1970s Mexico started to invest in biotechnology with the creation of important institutions such as the CINVESTAV (Centro de Investigación y Estudios Avanzados) of IPN (Instituto Politécnico Nacional) and the IBT (Instituto de Biotecnología) a part of the UNAM (Universidad Autónoma Nacional de México) among others [57,58]. In spite of establishing these research institutes, Mexico’s GERD increased slowly and unsteadily from 0.3% of its GDP in 2001 to 0.5% in 2014 [56,59]. The resources in Mexico for biotechnology have continued to be small, despite the creation, in 2013, of the government program PECiTI (Programa Especial de Ciencia y tecnología e Innovación). This program has as objective to promote biotechnology as one of the main principal science, technology and innovation fields. As a result of the small funding, it is not clear if this program has promoted HBT production in Mexico.

Argentina’s publication increase was 161% in HBT by the end of the period studied. The increase in Argentina’s production allowed it to continue to be ahead of Chile, Colombia and Cuba. Research in biotechnology started in Argentina in the 1980s and the government continued to support the field with its economic and scientific programs [19]. Argentina’s GERD was also steadily increasing from 0.4% of its GDP in 2001 to 0.6% in 2014 [59]. None of these factors seem to explain why Argentina had the periods of stagnation from 2001–2009. On the other hand, serious social-political conflicts in the country and economic difficulties in these periods could have affected Argentina’s levels of HBT publishing [60].

Chile’s publication increase was 156% in HBT by the end of the period studied. It is a considerable increase in spite of the limited budget Chile allocates to R&D. From 2006 to 2012 the increase in GERD was only 0.06% (from 0.3% of its GDP to 0.36%) [59]. This is in contrast to Chile’s policy to boost biotechnology, launched in 2004, that aimed to increase the GERD to 1% of GDP by the year 2006 [61].

Colombia’s publication increase was 400% in HBT by the end of the period studied. This is the highest publishing increase of all the countries examined in this study. This increase allowed Colombia almost to reach similar numbers of HBT papers as Chile at the end of the period. Colombia’s GERD is still small but it almost doubled from 0.11% of its GDP to 0.19% between 2001 and 2014 [56]. The country has developed its biotechnology policy over the years. It is promoting international participation in research in specific biotechnology fields [62–65]. These factors could have contributed to Colombia’s ability to boost its production in HBT.

Cuba’s publication increase was 123% in HBT by the end of the period studied. Its levels of HBT production are the lowest of the countries studied. This fits with the findings of Thorsteinsdóttir *et al*. in 2004 [43] and Debra in 2007 [66] where low scientific publishing in HBT and other scientific fields were reported. Publishing in academic journals is not a high priority of its government. Cuba rather emphasizes innovation, development and production of biotechnology products, where it has a strong track record. The strategy for biotechnology in Cuba is to use R&D as an input for product development that contributes to societal impacts instead of emphasizing knowledge generation reflected in publications as many other countries do [67]. The Cuban government also evaluates its R&D in HBT at the research centers, by measuring the impact of the results on the health of the population or the number of people that get healthy after treatments [68]. Lastly, another factor that has affected Cuba’s HBT publishing is the US embargo which has restricted Cuban publications in U.S. based journals [69]. The GERD as a percentage of GDP has fluctuated slightly in Cuba from 0.6–0.5% GDP from 2001–2013 and has apparently not affected Cuba’s HBT publishing [70].

Here we have examined the levels of HBT production in the six Latin American countries and tried to interpret our findings with reference to new policy initiatives, and the GERD as a percentage of GDP in the countries we studied. It is challenging to attribute patterns in knowledge production to any particular initiative in complex innovation systems. Our work, however, pointed to possible relationships between our results and particular policies in a number of cases that future research can address in more detail. It is clear that the three countries with the lowest levels of HBT publishing (Chile, Colombia and Cuba) are the smallest countries in the study in terms of population. Their capacity to publish in biotechnology could be better understood by other indicators such as the level of publishing per million inhabitants, which takes into account the smaller resources these countries have to allocate to biotechnology.

#### Health biotechnology papers per million inhabitants

**Fig 3:**
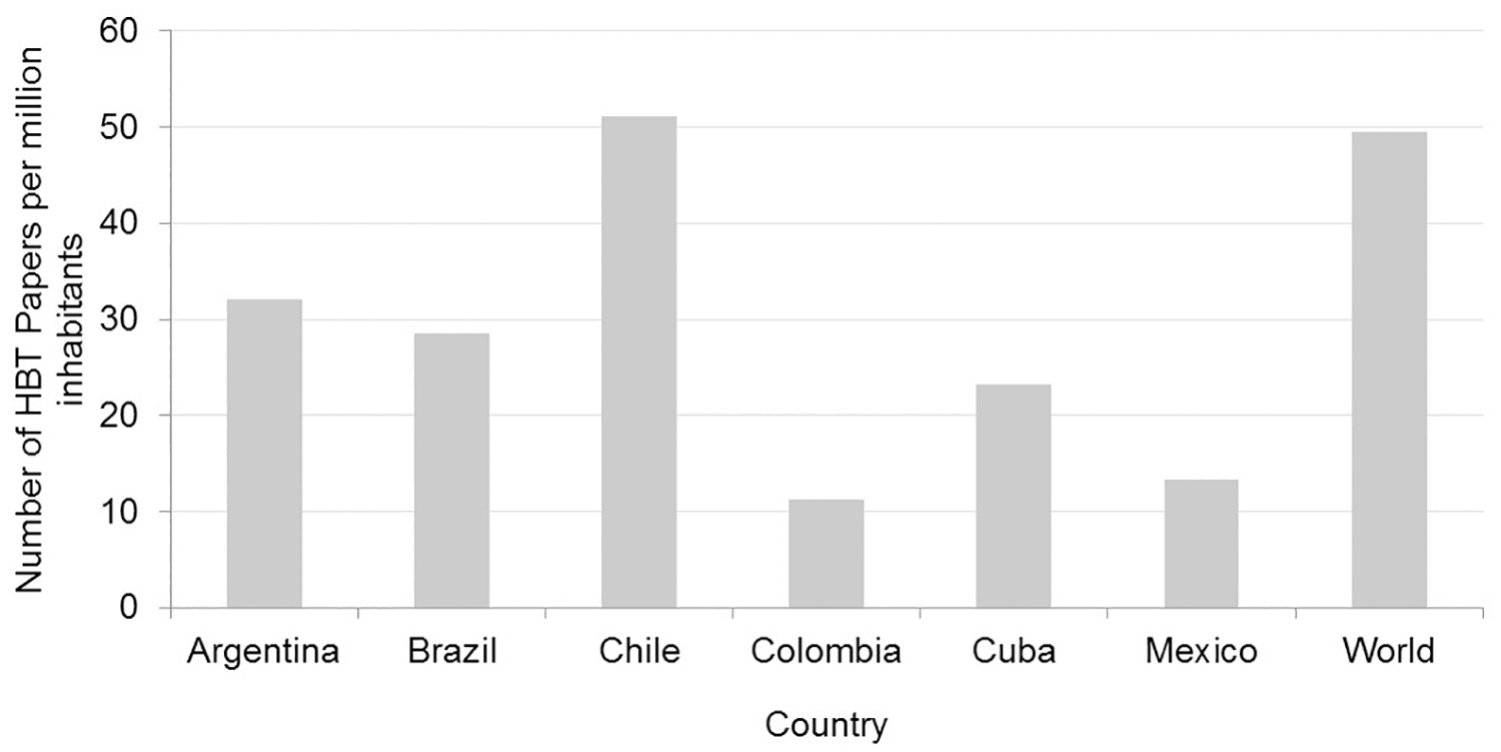
Number of publications in health biotechnology per million inhabitants, 2001–2015. Source: WOS data and United Nations Department of Economic and Social Affairs data [52,71].

Fig 3 depicts the number of HBT publications per million inhabitants as an effort to take into account the different sizes of the countries. We divided the total number of HBT publications from the years 2001–2015 for each country with the average population size in the focal countries [71]. The same process was done to estimate the number of HBT publications at the global level.

Almost none of the Latin American countries surpassed the world average of HBT publications per million inhabitants. The only exception is Chile, which has as many HBT papers per million inhabitants as the world average. Mexico and Colombia are the countries with the lowest average. The small countries Cuba and Chile demonstrated with this indicator that they are more productive in HBT publishing than larger countries such as Mexico.

#### The top 55 publishers in health biotechnology

**Fig 4:**
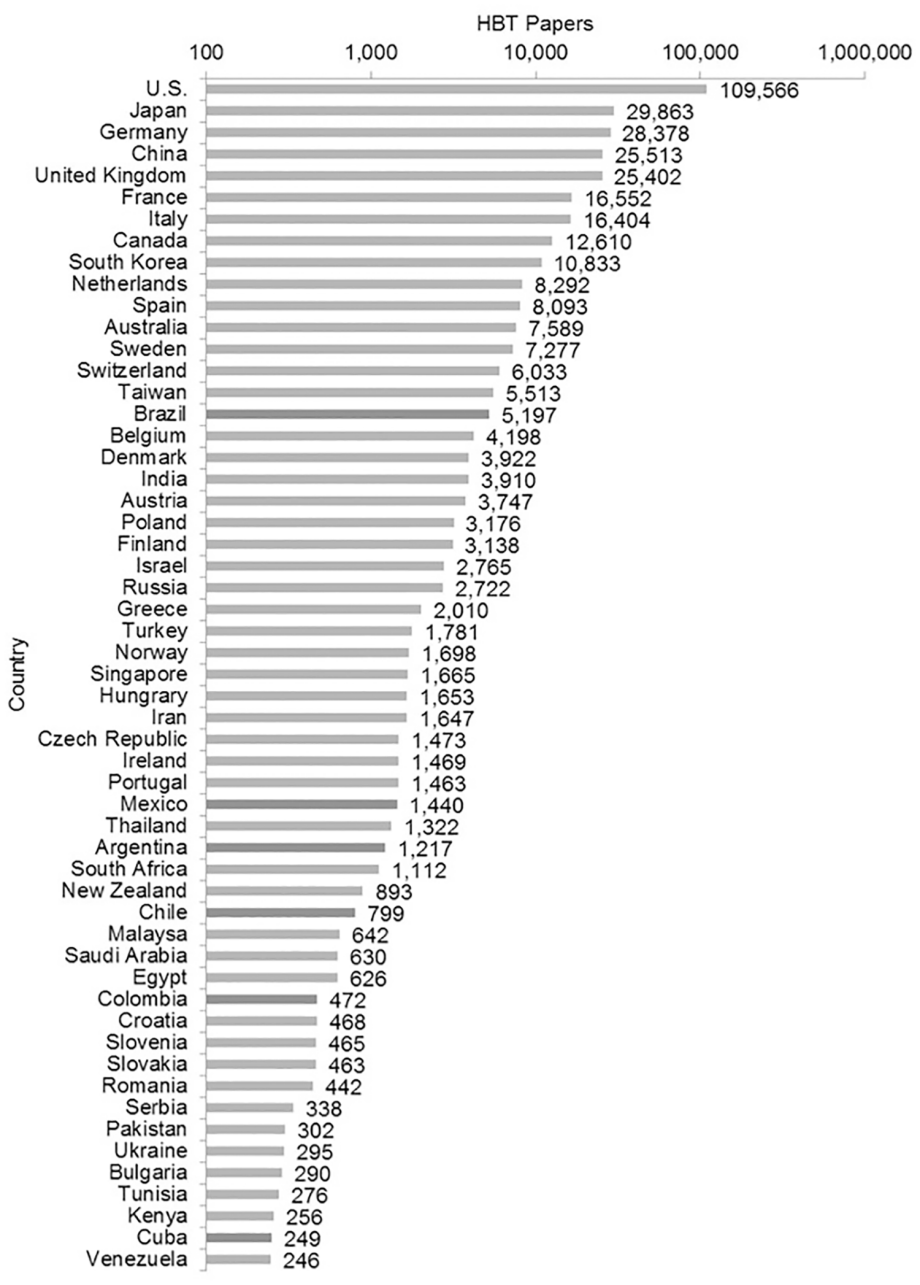
Top 55 publishers in health biotechnology, 2001–2015. Logarithm scale. Source: WOS data [52].

The two indicators discussed above present the trends in knowledge production of Latin American countries in HBT, but they do not present information on how these countries are positioned in the world in this field. To address this point we selected the 55 countries with the highest publishing in HBT globally and presented the data with logarithmic scale in descendent order (Fig 4). A logarithmic scale allows depicting values of different magnitudes from the countries’ HBT papers within a single graph. As happens in other science fields, such as nanobiotechnology and oncology, the U.S. is the leader in publishing in HBT, bypassing the next contender by more than 70,000 articles [72,73]

Brazil, Mexico and Argentina are the Latin American countries in our study that published more than a thousand of HBT papers in the period 2001–2015. Brazil surpassed small high-income countries, such as Denmark and Belgium and is the largest HBT publisher in Latin America.

Thorsteinsdóttir *et al* found in the period 1999–2002 that the average publishing growth in high-income countries was declining, while the publishing rate in emerging economies was increasing by more than 10%.[43]. A similar trend was found in a study on science productivity in general in developing countries [74]. We wondered if this trend was continuing in the 2012–2015 period and if it applied to our focal countries.

We examined the total HBT publishing of each year in the 2012–2015 period in the Latin American as well as other countries listed in Fig 4, to examine the trends in HBT publishing. The yearly growth rate was obtained by Eq (1):

$$G=\frac{P_{f}-P_{i}}{P_{i}}*100\%$$ (1)

Where *G* is the yearly growth rate percentage, *P*_*f*_ is the total amount of HBT publishing of a country in the latest year (2015), *P*_*i*_ is the total amount of HBT publishing of a country in the earliest year (2012). A negative *G* means that the HBT publishing rate is declining. A positive *G* means that HBT publishing rate is growing. A *G* = 0 means that HBT publishing rate is not changing over time in the country.

In Table 2, the trend in LMICs is similar as reported by Thorsteinsdóttir *et al*. [43] with countries such as the U.S., Canada and the United Kingdom having a negative yearly growth rate whereas most LMICs had positive rates. This, however, did not apply to all countries studied. While Brazil and Cuba reported a yearly growth rate of 25.2% and 10.8% in HBT in the 1999–2002 period [43], their yearly growth rate was -5.3% and -38.9% respectively in the period 2012–2015. In general, the yearly growth rate in LMICs was not more than 25% with the exception of Egypt and China. Chile was the Latin American country with the highest HBT publishing yearly growth rate and Mexico was number two.

**Table 2:** Yearly growth rate of papers in health biotechnology, 2012–2015 period.

Country	Yearly growth rate (%)
Egypt	48.4
China	38.8
**Chile**	**23.0**
South Africa	19.5
**Mexico**	**17.1**
India	15.3
Australia	12.2
Sweden	11.5
**Argentina**	**2.5**
Switzerland	-1.5
**Colombia**	**-2.2**
United Kingdom	-3.6
**Brazil**	**-5.3**
South Korea	-5.3
Denmark	-5.4
Canada	-8.4
U.S.	-13.2
Japan	-13.2
Germany	-13.8
Netherlands	-13.9
France	-15.8
Austria	-21.4
Spain	-24.6
Israel	-30.6
**Cuba**	**-38.9**

Bolded countries are the Latin American countries studied in this paper.

Source: Authors’ calculation based on WOS data [52].

These results suggest that HBT publishing in Latin American countries has not been growing as much in recent years as it did a decade ago. There can be a multitude of reasons for these results; for example, the decreasing general investment in research by some of the Latin American governments [56]; the instability of the markets as a result of the international financial crisis in 2009 [75] the 2012 financial slowdown in the Latin American countries [76–79], or political problems such as the armed conflict in Colombia [80] among others.

Although the number of papers of HBT gives important information about the trends in knowledge production in Latin American countries, to get a full picture it is necessary to examine also the citations received by the publications. Even though there is not a universally accepted indicator that reflects the impact, visibility or quality of a country’s research, the Average Relative Citations (ARC) is an indicator which is now adopted in diverse studies as evidence of quality research in a particular field or country [43,81–85]. Another indicator is the Relative Citation Ratio (RCR), recently proposed [86]. This indicator quantifies the influence of a research article by the use of co-citation networks. The RCR has been used to demonstrate correlations between articles that are relatively influential and opinions of subject matter experts in biomedical research [86] and has been adopted by the National Institutes of Health.

To calculate the ARC of each country we applied Eq (2). Where *ARC* is the Average Relative Citations Indicator, *P*_*c*_ are the number of papers in HBT published by a country in the 2001–2015 period, *C*_*c*_ are the total amount of citations received by each *P*_*c*_ paper in HBT during the subsequent two years of its publication (with the exception of the years 2014 and 2015 that are years that we could not obtain complete information), *P*_*w*_ are the number of papers in HBT published in the world in the period 2001–2015, *C*_*w*_ are the total amount of citations received by each *P*_*w*_ paper in HBT during the subsequent two years of its publication.

$$ARC=\frac{\frac{C_{c}}{P_{c}}}{\frac{C_{w}}{P_{w}}}$$ (2)

An *ARC* value above 1 for a country means that the publications of the country in that field are cited more often than the world average. An *ARC* value below 1 for a country means that its publications in HBT are not cited as often as the world average [87].

To calculate the RCR of each country we used the iCite beta version [88]. We obtained a Pubmed manuscript IDs (PMIDs) list on HBT for the six Latin American countries at the Pubmed database of the National Center for Biotechnology Information (NCBI) by applying the same search criteria to identify HBT papers in WOS used in this study. The value obtained is the Weighted RCR, which is the sum of the RCRs values in a group of papers. A highly influential group of papers will have a higher weighted RCR value than the total number of publications, while a below-average group of papers will have a weighted RCR below the number of publications [88].

**Fig 5:**
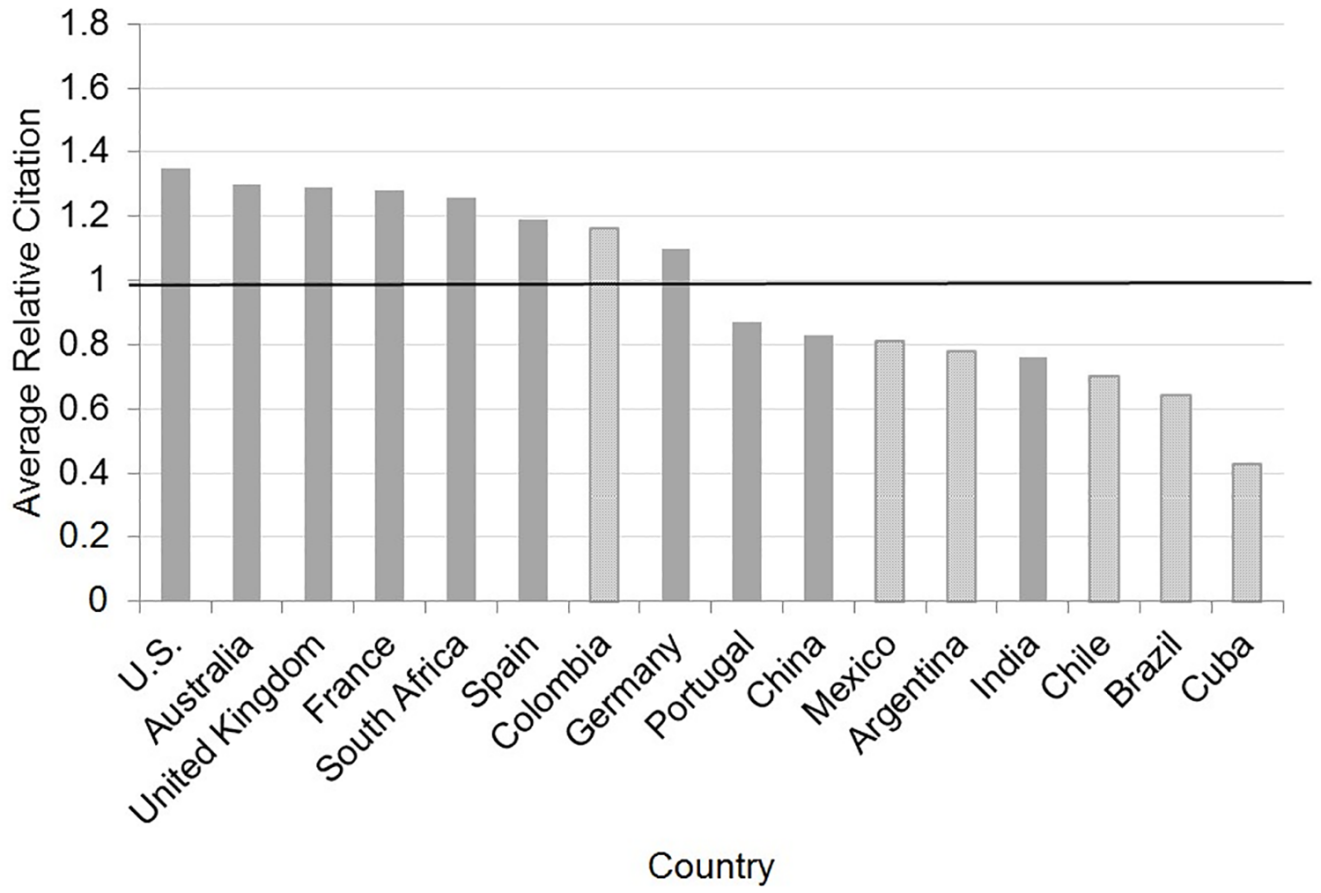
Average relative citations of Latin American countries and other selected countries in health biotechnology, 2001–2015. Source: WOS data [52].

The Latin American countries are not reaching the world average level in ARC in HBT, with the exception of Colombia (Fig 5). It has a higher ARC than even countries with a longer tradition in biotechnology such as Germany. When we examined the case of Colombia, we observed that there are three articles during the period examined that were highly cited and represented 20% of the total amount of citations. These articles involved international collaboration, so the high ARC in Colombia could be attributed to the capacity of Colombian researchers to participate in important international research projects. Gonzalez-Brambila *et al*. [74] provided some evidence of how important international research projects seem to impact the visibility of astrophysics and astronomy publications in Chile. In general, the observation that some Latin American countries have similar or higher ARC than shown by earlier research [43] may indicate that Latin American countries are not only increasing their knowledge production in HBT but also the visibility and impacts of their papers.

The Latin American countries studied are below the average level in RCR in HBT. None of the countries selected could reach a higher weighted RCR value than the total publications (Table 3). Brazil was the country that had the highest weighted RCR/publications ratio but it was far under the number of publications.

**Table 3:** Relative citations ratio of Latin American countries and other developing countries in health biotechnology, 2001–2015.

Country	Publications	Weight RCR
Argentina	676.00	104.36
Brazil	7445.00	3149.43
Colombia	221.00	41.10
Chile	613.00	95.06
Cuba	81.00	3.20
México	1203.00	192.05

Source: iCite beta version [88]

### Main actors in health biotechnology publishing in the Latin American countries

We classified the HBT papers in each of the focal countries by publishing actors. The publishing actors classification was made based on the authors’ declared affiliations: University, which can be public or private; Government, which are the government research centers, institutes and the research departments at government secretaries; Company, which are the firms dedicated to commercializing HBT products and typically have R&D departments; Clinics & hospitals, which can be public or private health care centers, clinics and hospitals; Finally, Others, which are non-governmental organizations, foundations, public or professional associations among others.

The affiliation of each author of the HBT papers in the period was classified, according to their declared sector, and then we calculated the percentage of the sectors’ involvement based on the total amount of papers in HBT. Many of the papers include authors from different sectors and we decided to count all the sectors involved for each paper. Therefore the total percentage from the five sectors exceeded 100%.

**Fig 6:**
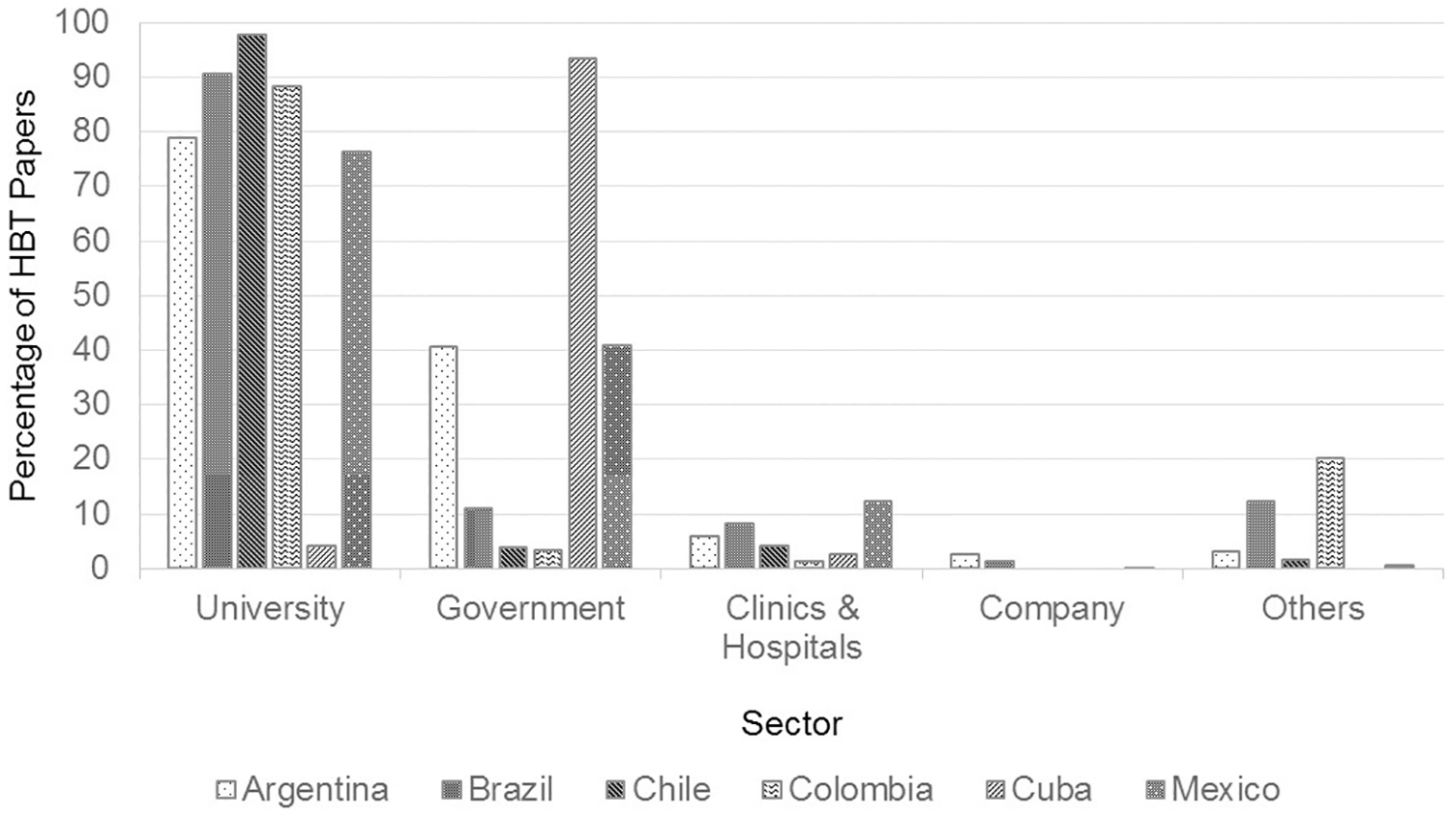
Percentage of papers in health biotechnology, per sector, 2001–2015. Source: WOS data [52].

The main knowledge production in HBT is done by the university sector (Fig 6). This fits the model typically implemented in Latin America in which Universities’ principal function is knowledge production [89,90]. Universities in most of the Latin American countries focused on are involved in more than 70% of the HBT papers, with the exception of Cuba (less than 10%). The relatively small emphasis on universities as knowledge producers in Cuban HBT has been analyzed before by Thorsteinsdóttir et al. for the period 1991–2002 [43]. The Cuban HBT knowledge production is primarily carried out at governmental research centers, while universities have a higher relevance for human resource training [91].

Another interesting notion is the contribution of graduate studies to HBT knowledge production by universities. It is common for university programs in the Latin American countries we studied to require that their graduate students publish an original scientific paper in order to obtain their degrees, and this strategy may help to increase the knowledge production. Unfortunately the WOS database does not provide data on graduate students involvement in the papers it lists.

The participation of the governmental sector is not only relevant to the Cuban case. In Brazil, Mexico and Argentina the governmental sector publishes more than 20% of the HBT papers. The government organism for science and technology in Mexico, CONACYT (Consejo Nacional de Ciencia y Tecnología), and its Argentinian homologous, CONICET (Consejo Nacional de Investigaciones Científicas y Técnicas), have a structure of grants for graduate students and researchers at governmental research centers, where they are evaluated based on the number of their publications in international peer reviewed journal [92,93]. This can explain the relatively high percentages of the government sector in the HBT publishing in those countries.

There is a low participation in HBT publishing by the clinics and hospitals sector in the countries examined. The highest participation was for Mexico with 12% of the HBT papers. This shows that research by hospitals, clinics and other health care centers in HBT that lead to publications in indexed journals, has been limited in most Latin American countries, as is generally the case in other LMICs [43]. Our research also showed that Mexican hospitals have been lessening their emphasis on local journals and publishing more in international mainstream journals. This change increased the visibility of Mexican hospitals in databases, such as WOS, PubMed and Scopus. Although there are efforts to register local journals in indexed peer reviewed databases, there are many local journals that are not internationally indexed, in which hospitals and clinics in Latin American countries seem to continue to publish.

In almost all the focal countries there is a low HBT publishing participation of companies with less than 4% of the papers. This fits with the notion that the traditional function of firms is to generate goods and services but not to emphasize scientific publications.

Colombia has a peculiarly high participation of the ‘Others’ in HBT publishing whereas its government has the lowest participation rate in HBT publishing compared to the focal Latin American countries. In Colombia there is a non-government foundation, FIDIC (Fundación Instituto de Inmunología de Colombia), which partly explains this high rate and is responsible for 6.5% of the HBT publishing.

### Main health biotechnology subfields of the focal countries

In order to explore what are the main areas that the Latin American countries publish in, we classified the papers into research subfields of HBT and calculated their specialization indices. The specialization index is an indicator that expresses the ratio of research of a country in a specific field, normalized with the ratio of research of the whole world in that field. A specialization index above 1 indicates that the country publishes in a specific field more than the world does and therefore the country is specialized in that field. A specialization index under 1 indicates that the country publishes in a specific field less than the world does and therefore the country has limited interest or capacity in that field.

The specialization index was obtained by Eq (3) that is:

$$S_{i}=\frac{\frac{S_{c}}{T_{c}}}{\frac{S_{w}}{T_{w}}}$$ (3)

Where *S*_*i*_ is the specialization index, *S*_*c*_ is the total amount of a country publishing in a specific sector in the studied period, *T*_*c*_ is the total country publishing in the studied period, *S*_*w*_ is the total amount of the world publishing in a specific sector in the studied period and *T*_*w*_ is the world publishing in the studied period. In addition, we carried out data mining to the papers that were categorized in the HBT subfields with a specialization index over 1 to identify the most frequent research topics.

Table 4 shows that the Latin American countries promoted three main HBT subfields: tropical medicine, infectious diseases and parasitology. The high amounts of parasites, as well as tropical and infectious diseases found in tropical climate, coupled with the high infection rate related to the sociodemographic profiles of the Latin America countries, seem to fuel this research emphasis. This reflects that Latin American countries are attempting to use HBT knowledge to understand and obtain solutions to their local and endemic health problems. The main topics in parasitology are related to the study of the metabolism and control of parasites. The species most studied are *Trypanosoma*, *Entamoeba* and *Plasmodium*. These species have a high prevalence in all the Latin American countries [94,95].

**Table 4:** Subfields of health biotechnology in the 2001–2015 period with specialization indices > 1.

Country	Argentina	Brazil	Chile	Colombia	Cuba	Mexico
Health biotechnology subfield						
Biochemical and Molecular Biology					1.16	
Immunology	1.24	1.17		1.97	1.44	1.58
Pharmacology Pharmacy					1.58	
Genetics Heredity				1.32		
Biotechnology Applied Microbiology	1.01				3.09	1.22
Microbiology	1.89	1.73		2.25		1.53
Endocrinology Metabolism	1.61		1.60			
Research Experimental Medicine				1.10	1.57	1.42
**Tropical Medicine**	4.29	15.90	1.04	20.24	10.02	3.18
**Infectious Diseases**	2.43	2.73	2.04	4.22	1.84	2.16
**Parasitology**	4.42	10.47	9.22	9.00	5.20	5.78
Dentistry Oral Surgery Medicine		4.16	1.96	3.83		1.15
Toxicology	1.42					2.02
Reproductive Biology	3.40		5.92			1.87
Obstetrics Gynecology	2.58	1.36	4.62		1.35	1.62
Virology	1.65	1.21		1.65		1.24
Developmental Biology	4.13		7.90			1.74
General Internal Medicine	1.76		1.99	3.76	4.81	1.45
Hematology					1.14	
Life Sciences Biomedicine Other Topics			1.04	1.16		1.49

The health biotechnology subfields in which all the main Latin American countries have specialization indices > 1 were bolded

Source: Authors’ calculation based on WOS data [52].

In tropical diseases, there is intense research related to leishmaniosis and dengue fever. There have been several national and international programs and plans to study, control or eradicate these diseases [96]. Finally, in the infectious diseases subfield, there are intense research efforts in *Candida* spp., *Mycobacterium* spp., HIV (human immunodeficiency virus) and HPV (Human papillomavirus). These infectious agents are in a significant portion of the population in Latin American countries [97].

Others HBT subfields which are emphasized by almost all the Latin American countries are gynecology and obstetrics, immunology, and general internal medicine. We found that these three subfields were closely related to an emerging non-communicable disease burden increasingly common in Latin American population, such as obesity, hypertension, diabetes, breast and cervical cancer, as well as communicable diseases such as HPV and HIV.

The fact that Latin American countries seem to focus their biotechnology research on their national health problems can be connected to governmental prioritization. In Latin American countries governments are the main funders of research. Budgets are allocated primarily to those research groups that align themselves with the national science and technology plans, and seek to remedy national problems through science [98–101].

### Patterns of health biotechnology collaboration of the focal nations

#### International collaboration

To examine the trends in international collaboration, we identified the HBT paper of the focal Latin American countries that had at least one co-author with an address in another country. This portion was divided with the total amount of HBT publishing to obtain the percentages of papers in international collaboration.

**Fig 7:**
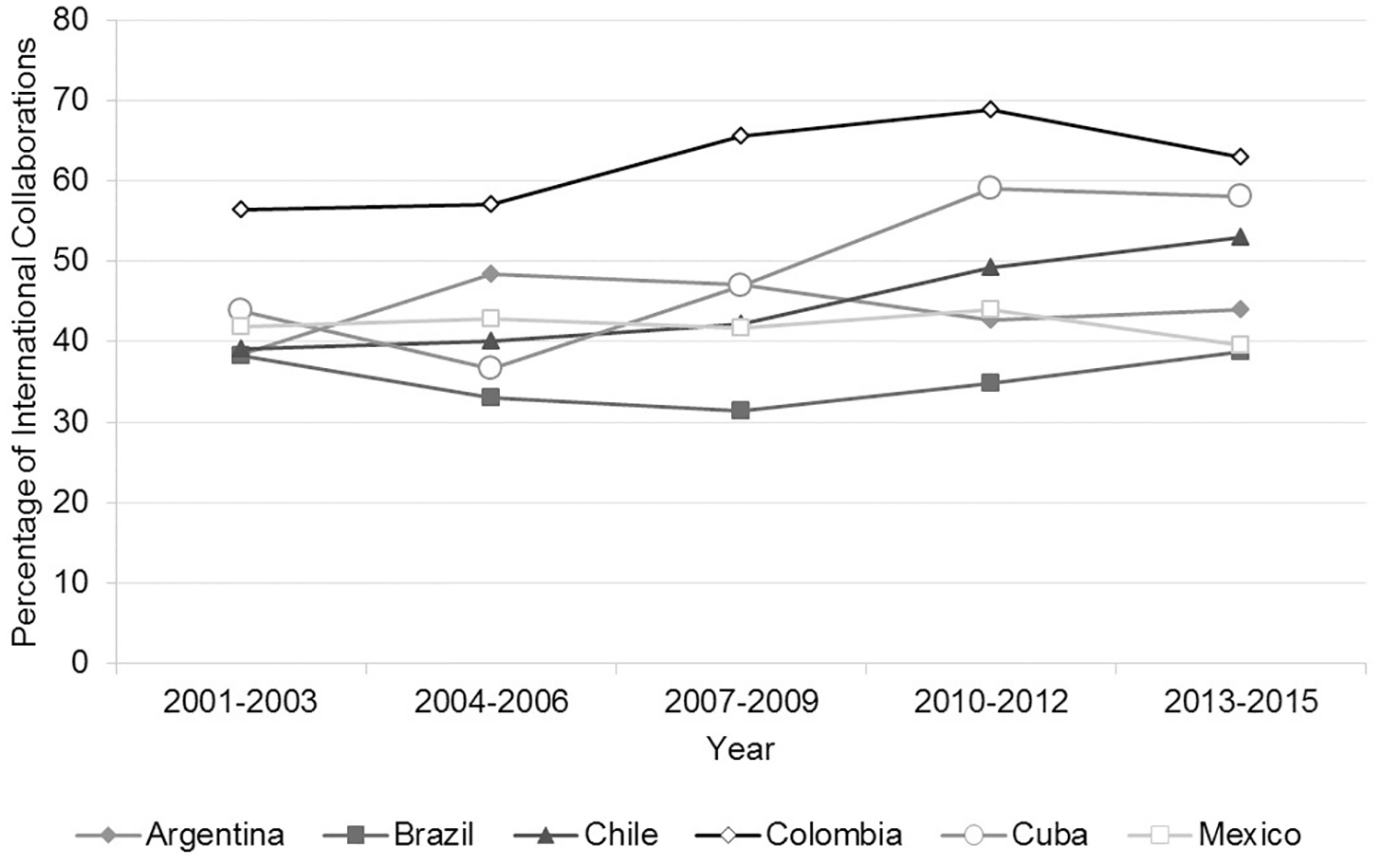
Rate of international collaboration in health biotechnology, 2001–2015. Source: WOS data [52].

The average international collaboration rate in the Latin American countries we studied during the period was 45% (Fig 7). Colombia had the highest level of international collaboration in HBT publishing. In the early 2000s, Colombia had nearly 70% of its papers in international collaboration. This percentage has been diminishing to 60% at the end of the period, but it continues to be the most active country in international collaboration. The Colombian government has, since the 1990s, implemented strategies that promote international collaboration in science and technology [102,103]. This could explain why international collaboration in Colombia is the highest among the Latin American countries. Another potential is that Colombia may need international collaboration in order to gain access to specialized research infrastructure or expertise in the field.

In Cuba, the percentage of international collaboration has been growing during the period from 44% to 58% of its HBT papers. HBT is one of the strongest biotechnology sectors in Cuba, and researchers from the island may be sought after partners [30,66,104]. This could explain the observation that the Cuban percentage of international collaboration in the period, has been increasing gradually in HBT publishing. In 2016, Cuba signed new technology cooperation agreements with China to promote the exchange of HBT products, therapies and R&D [105]. We can expect that Cuba’s rate of international collaboration increases in the near future with more international ties being established.

Mexico’s international collaboration rate changed the least of the countries studied. It started with 41% and it finished the period with almost the same percentage. Mexico includes an emphasis on international cooperation in its science, technology and innovation policies and CONACyT has set up a special section, the PCI (Portal de Cooperación International), where international collaboration is promoted with grants and the agreements with international universities [106]. Mexico can also be a partner in the European Union’s Horizon2020 program [107]. It has a special funding for international cooperation in science and technology administered by CONACyT, known as FONCICYT (Fondo de Cooperación Internacional en Ciencia y Tecnología del CONACyT). The problem is that the government is constantly decreasing CONACyT’s funding so it is only available to a small portion of the country’s researchers [108].

Argentina’s international collaboration in HBT publishing has not changed much for the period studied. It started with 39% of its paper published in international collaboration at the beginning of the period and ended with 42%. Argentina’s largest emphasis on international collaboration in HBT was in the 2004–2006 period with 49% of the papers involving international collaboration. In these same years, Argentina had the lowest GERD [56]. It diminished from 0.45% of the GDP to less than 0.34%, which may indicate an economic necessity to obtain international funding to continue Argentina’s R&D in HBT during those economic challenges. The strategy of Argentina to harness international collaboration for knowledge production continues nowadays. In 2011, it inaugurated the research center PCT (Polo Científico Tecnológico) with national and international funding. This research center includes a unit of the ICGEB (International Centre for Genetic Engineering and Biotechnology) that is focused on advancing research and training on genetic engineering and biotechnology [98].

Brazil has applied different policies with regards to international collaboration during the period studied. In the first half of period, Brazil tried to generate its own science and technology development in strategic sectors such as biotechnology [100] with policies and laws that encourage the domestic collaboration rather than international collaboration [109]. This could explain the decreased levels of international collaboration in HBT knowledge production in the period 2001–2009. After 2010, Brazil changed its strategy and aimed at developing science and technology by relying on international scientific collaboration. For example, in 2014 Brazil established the OIB (Observatory of Innovation in Biotechnology) with the participation of the European Union [110]. This could explain why Brazil increased its emphasis on international collaboration and ended with almost 40% of its papers published in international collaboration in the 2013–2015 period.

Legislative constraints and periods of instability due to frequent changes in government in the early years of the period studied induced budgetary delays, disrupted continuity in funding and discouraged venture capital investments [25,111]. In the early 2000s, around half of the Brazilian HBT companies were newly founded micro-companies or small-sized firms with little innovation, patenting or new knowledge production [112,113]. At that time, domestic university-industry collaboration was encouraged, nonetheless these alliances were oriented to obtain existing HBT innovation rather than to develop HBT knowledge that could be harnessed for future innovation [25]. In 2004, the Brazilian government placed emphasis on encouraging linkages between the public research system and companies with, for example, the Innovation Law [25,111]. This legislative change could have had an impact on the entrepreneurial culture by encouraging HBT knowledge production in the latest years studied.

Chile also increased its international collaboration in HBT during the period studied. It increased from 39% of its HBT papers in the beginning to 52% at the end. Over the period, Chile has signed various agreements promoting biotechnology collaboration [114]. These agreements are promoted by its CONICYT (National Commission for Scientific and Technological Research) that has as one of its many objectives to exchange scientific knowledge with international parties.

The Latin American countries studied, except for Brazil, all aimed their public policies at strengthening international collaborations to generate scientific knowledge in HBT. Brazil was the only country that had an uneven emphasis on international collaboration and it decided to strengthen the domestic research collaboration in HBT rather than international collaboration. The same trend has been observed in other science sectors such as in nanotechnology [115].

#### Main collaborators of the Latin American countries

Table 5 lists the principal countries that collaborate with the six Latin American countries in HBT. The numbers indicate the percentages >1% of their HBT papers that are published in collaboration with each country.

**Table 5:** Main collaborators of the focal Latin American countries in health biotechnology, 2001–2015.

Country	Argentina	Brazil	Chile	Colombia	Cuba	Mexico
	HBT %	HBT %	HBT %	HBT %	HBT %	HBT %
Brazil	6.6		3.2	6.3	4.0	4.2
U.S.	18.1	17.7	17.4	32.3	6.4	25.8
France	5.3	4.5	4.3	10.9	4.6	4.2
United Kingdom	3.4	3.7	4.1	4.2	5.2	3.3
Germany	3.6	3.5	4.9	4.2	7.5	3.4
Italy	4.7	2.7	5.2	3.7	7.5	1.7
Canada	2.4	2.6	1.4	4.4	1.7	2.9
Spain	6.6	1.9	7.9	13.5	13.9	6.1
Argentina		1.7	3.6	4.4	1.7	1.6
Netherlands	1.4	1.4	2.1	4.2	4.6	1.8
Portugal	1.1	1.4			1.7	
Mexico	1.9	1.3	2.0	7.0	4.6	
Belgium	1.4	1.2		2.3	1.7	
Japan	1.9	1.1	1.9	3.9	2.3	1.9
Australia		1.2	2.3	3.5	1.7	1.6
Colombia	1.5		2.1			2.0
Chile	2.3			3.9	2.3	1.1
Poland			1.5	3.5		
Peru	1.2			4.6		1.2
Venezuela					3.8	

Source: Authors’ calculation based on WOS data [52].

The U.S. is the principal collaborator in HBT in five of the Latin American countries. Cuba is the only one that has Spain as its main collaborator instead of the U.S. Spain ranks highly as a collaborator for all the Latin American countries we studied except for Brazil. Colombia had the highest percentages of collaboration with many of the countries. This was expected as Colombia has the highest levels of international collaboration in the period. It is noteworthy that several European countries are heavily involved in research collaboration in HBT with the Latin American countries studied. The European Union, and some of its member states, have made several science collaboration agreements, in different fields including biotechnology, with all the Latin American countries studied [116,117]. When we analyze international collaboration within Latin America, Brazil figures as the main collaborator. The Brazilian collaboration trend in HBT is similar to other science sectors, such as physics, biology and chemistry [118]. Nevertheless, the collaboration in HBT is less than 10%. The low collaboration levels among Latin American countries in the period, suggests that these countries may not see potential benefits of strengthening the Latin American collaboration in HBT.

The six Latin American countries co-authored papers, in South-South collaboration, on several themes in HBT in the period studied. The three main HBT themes they addressed in South-South collaboration were: research on the papilloma virus/cancer; Human Immunodeficiency Virus (HIV) /immunodeficiency diseases; and *Trypanosoma cruzi*/parasites.

An example of South-South collaboration in HBT is the Brazil-Mexico-Colombia collaboration on the treatment, diagnostic, points of prevalence, genotype distribution, and incident of infections of papillomavirus in Latin America. This makes sense when statistics report 36,000 deaths by cervical cancer linked to human papillomavirus (HPV) in the Latin American region in 2012 [119]. This number is expected to increase by 45% in 2030 if the current tendency continues. The most recent article on this theme was published in 2015 and was on the efficacy of a bivalent vaccine against oncogenic papillomavirus 16/18 by the Papilloma Trial against Cancer In young Adults (PATRICIA) study group [120]. This is an international group financed by GlaxoSmithKline Biologicals for the evaluation and monitoring of the HPV-16/18 AS04-adjuvanted vaccine trial. The development of this adjuvant vaccine trial is important because approximately 70% of cervical cancer cases in the region are attributed to high-risk HPV-16 and -18 [119]

Teams from Brazil-Mexico-Argentina publish most of the South-South collaboration on HIV. This work is focused on the epidemiologic and molecular characterization of HIV Type 1 (HIV Type 1 is common in Latin America), drug resistance mutation profile in HIV-patients and the effect of new inhibitors in the HIV replication, among other topics. Nowadays, HIV is an important health problem with on average over 85.000 new infections a year (2015 estimations) in the Latin American and Caribbean region [121]

Teams from Brazil-Colombia-Chile have also collaborated on research on *Trypanosoma cruzi* the parasite that causes Chagas disease, that is prevalent on the continent. The research has focused on genetic analysis, proteomics of trypanosomatids and metabolism regulation. The World Health Organization (WHO) estimates that there are between 6 and 7 million people infected with *Trypanosoma cruzi* in the world, most of them in Latin America [122].

#### Domestic collaboration

**Fig 8:**
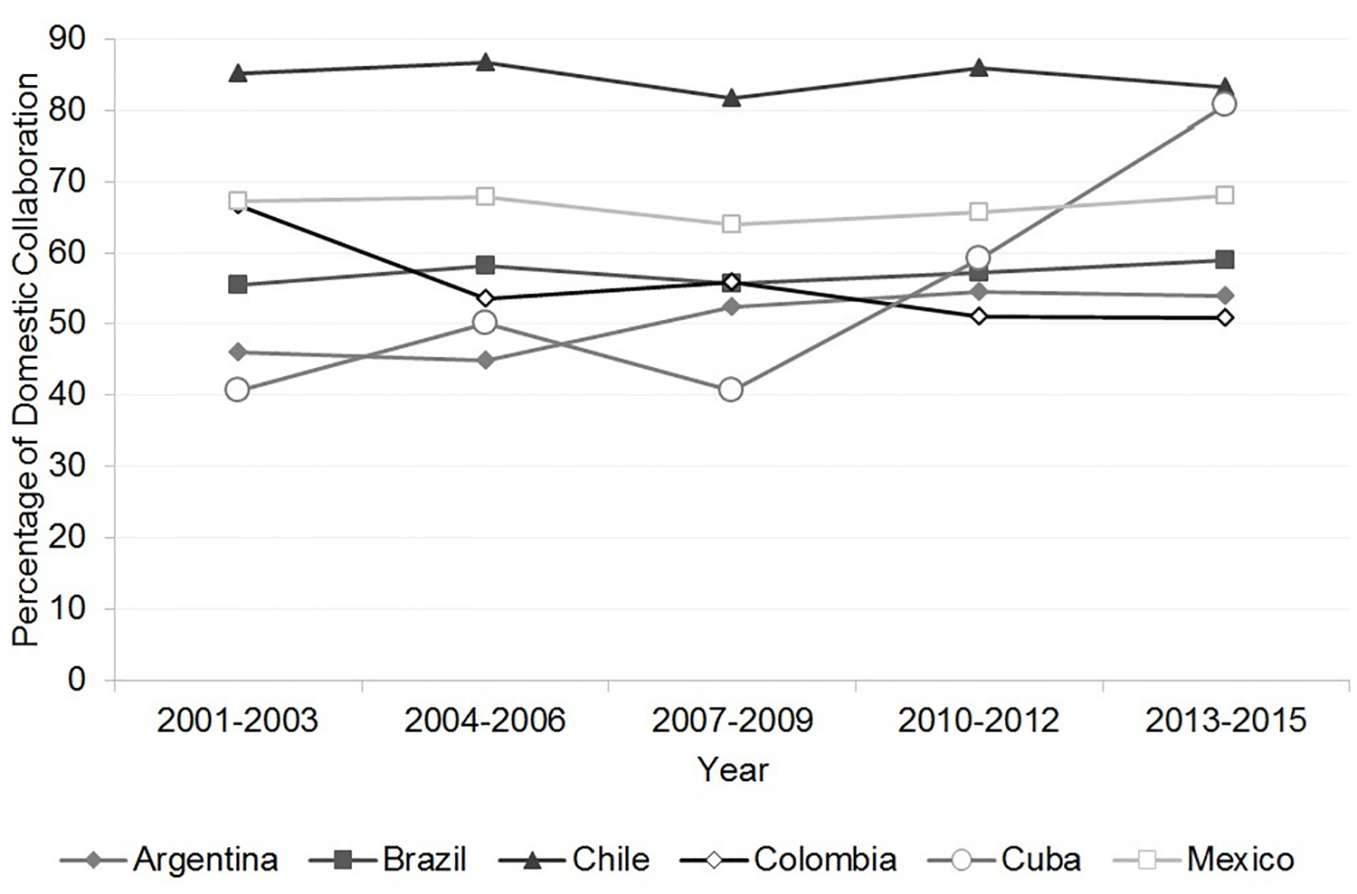
Rate of domestic collaboration in health biotechnology, 2001–2015. Source: WOS data [52].

We included an examination of the domestic collaboration of the Latin American countries in our study to complete the collaboration panorama. Domestic collaboration was defined as the ratio of co-authorships, involving more than one domestic institution, divided by the total number of HBT papers of the country (Fig 8).

All the Latin American countries had relatively steady domestic collaboration, with the exception of Cuba. Chile and Mexico maintained the highest percentages with more than 69% of their HBT papers produced in domestic collaboration during the period studied. These countries had since the early 2000s implemented strategies and policies that aimed at generating domestic linkages in several science sectors, including biotechnology [22,99,123].

Cuba had 40% of its papers produced in domestic HBT collaboration at the beginning of the period we studied and finished with 80% at the end. An emphasis on domestic collaboration has been an ongoing feature of Cuba’s biotechnology development from the very beginning of the development of the sector [30]. It could have been reinforced by the creation of the Group of Biotechnology Industries and the Pharmaceutical BioCubaFarma in 2012. This group was made by the integration of the universities, the entities belonging to the Scientific Pole and the business group QUIMEFA [91].

Colombia had around 50% of its HBT papers published in domestic collaboration. This percentage was steady for most of the period of the study. Colombia increased its domestic collaboration in HBT in the 2001–2003 period and then returned to the 50% in the next period studied. This increase in domestic collaboration coincided with the lowest levels in the Colombian R&D expenditures that were reported for the years 2001 and 2002 [56]. It is an indication that the increase in the domestic collaboration may have been to offset the shortcoming in budgets and not because of changed policies for science and technology in Colombia [101,124,125].

Brazil maintained almost the same percentage of domestic collaboration in HBT during the period studied. Its government enacted in 2004 the Innovation Law. This law affirmed that its science institutes should form technological innovation nuclei with other national institutes, to protect and own their creations, and promote licensing, innovation and other forms of technology transfer produced by Brazil’s research entities [126].

Argentina increased its domestic collaboration in HBT modestly during, the studied period. The country promulgated in 2001 the Law on science, technology and innovation. This law encourages domestic collaboration with the establishment of the CICYT (Interagency Council for Science and Technology), which is composed of all the national science institutions.

## Conclusions

Based on the research reported in this paper we conclude that: Firstly, the Latin American countries studied increased their HBT production in the 2001–2015 period. Their HBT production was 1.9% of the total amount of health biotechnology production in the world in 2001 and increased to 3.5% in 2015. This increase has been larger than their increase in the total amount of the biotechnology production, that according to the Scimago Journal & Country Rankings increased from 3.1% in 2001 to 3.6% in 2015 [127]. The same applies to global science production that according to Scimago increased from 2.5% in 2001 to 3.7% in 2015.

Furthermore, many of the countries studied had higher growth rates in HBT than the leaders in biotechnology from high-income countries, which could mean that the gap in HBT is likely to be reduced between the Latin American countries and those leading countries. This trend had been observed recently in science productivity in general in LMICs, including in some countries in Latin America [74]. To maintain this trend, it is necessary that governments continue to promote biotechnology research in Latin America. We have provided some evidence of how government programs and investments seem to impact the growth rate in HBT publishing. The best example is Brazil, whose government has consistently promoted science programs and steadily increased the GERD during the period studied. Brazil has become one of the leading countries in HBT and is overtaking some smaller European countries.

Secondly, the study reveals that Latin American ARC and RCR levels in HBT are lower than the World average with the exception of Colombia, in the case of ARC. This means that the international scientific community does not pay attention to the contributions of Latin American countries in HBT to the same degree as to contributions from European countries or the US.

Thirdly, this study provides evidence that universities are the main producers of HBT publishing in all the Latin American countries studied, except in Cuba, as has been proposed in previous studies on science production on the continent [25,128,129]. In Cuba the government sector continues to be the main contributor to HBT. This difference between Cuba and the other Latin American countries is partly because Cuba’s government institutions have the duty to create innovations, not scientific publications, and universities have the primary role to provide human resources training [91]. In the other Latin American countries a heavier emphasis is placed on universities to be knowledge producers, in addition to provide training [90]. We found evidence in Argentina that the participation of government sector in HBT production is gradually increasing, where the government participation in the 2001–2015 period grew from 36% to 53%. This trend could be explained in part by the government's strategies to promote university-government-industry collaboration with the aim of increasing innovation and knowledge production on issues of national interest [130]. The opposite case has happened in Chile, where the participation of the government sector fell from 11% to 4%. Although Chile has promoted collaboration on a modest scale in biotechnology [129]. the collaboration is highly focused on agro-and pisciculture biotechnology rather than HBT [27,61,99].

Fourthly, apparently, every Latin American country we focused on has had similar international collaboration strategies. In general, there have been no significant changes in the international collaboration in HBT knowledge production of the six countries over the period studied measured by co-publications. Our results agree with the results of Confraria and Vargas that analyzed knowledge production in Latin America in a number of scientific fields by using a combination of bibliometric, social network and econometric techniques and focused particularly on the relationship of research departments which belong to universities, research institutes or government agencies, with the industry sector in the 2004–2013 period[130]. Their data did not show much change in the rate of international collaboration for the period studied, but they highlighted that countries with small science systems relied more heavily on international collaboration than countries with larger science systems. International collaboration in HBT may represent to Latin American countries opportunities to achieve their goals faster of promoting health, innovation, and competitiveness, as has been observed in other scientific fields [118,131–134].

Finally, the subfields in health biotechnology where the Latin American countries specialized in are those needed in order to address local health problems. This strategy has been applied in health and other fields with positive results [5,135,136]. In HBT this strategy could bring innovations that may contribute to solving national health problems, in addition to bringing welfare and economic growth to the Latin American countries.
